# First Evidence of Anti-*Plasmodium vivax* (Plasmodiidae): Activity of the Essential Oil and 6-Ishwarone Isolated from *Piper alatipetiolatum* Yunck. (Piperaceae)

**DOI:** 10.3390/biomedicines13112785

**Published:** 2025-11-14

**Authors:** Glenda Quaresma Ramos, Renata Galvão de Azevedo, André Correa de Oliveira, Maria Luiza Lima da Costa, Felipe Moura Araujo da Silva, Ingrity Suelen Costa Sá, Gisely Cardoso de Melo, Stefanie Costa Pinto Lopes, Gemilson Soares Pontes, Sergio Massayoshi Nunomura, Rita de Cássia Saraiva Nunomura, Rosemary Aparecida Roque

**Affiliations:** 1 Centro Multiusuário para Análise de Fenômenos Biomédicos, Universidade do Estado do Amazonas, Manaus 69065-001, Amazonas, Brazil; gq.ramos01@gmail.com; 2 Programa de Pós-Graduação em Ciências Biológicas-Biofísica, Instituto de Biofísica Carlos Chagas Filho, Rio de Janeiro 21941-902, Rio de Janeiro, Brazil; 3 Programa de Pós-Graduação em Imunologia Básica e Aplicada, Universidade Federal do Amazonas, Manaus 69080-900, Amazonas, Brazil; renatagalv28@gmail.com; 4 Laboratório de Virologia e Imunologia, Instituto Nacional de Pesquisas da Amazônia, Manaus 69067-375, Amazonas, Brazil; pontesbm1@gmail.com; 5 Laboratório de Controle Biológico e Biotecnologia da Malária e da Dengue, Instituto Nacional de Pesquisas da Amazônia, Manaus 69067-375, Amazonas, Brazil; luiza3646@hotmail.com (M.L.L.d.C.); rosebio1996@yahoo.com.br (R.A.R.); 6 Central Analítica, Centro de Apoio Multidisciplinar, Universidade Federal do Amazonas, Manaus 69080-900, Amazonas, Brazil; 7 Programa de Pós-Graduação em Entomologia, Instituto Nacional de Pesquisas da Amazônia, Manaus 69067-375, Amazonas, Brazil; 8 Coordenação de Tecnologia e Inovação, Instituto Nacional de Pesquisas da Amazônia, Manaus 69067-375, Amazonas, Brazil; felipemourams@gmail.com; 9 Instituto de Saúde e Biotecnologia, Universidade Federal do Amazonas, Campus Coari, Coari 69460-000, Amazonas, Brazil; ingrity.scosta@gmail.com; 10 Fundação de Medicina Tropical Dr. Heitor Vieira Dourado e Universidade do Estado do Amazonas, Manaus 69050-085, Amazonas, Brazil; cardosogisely@gmail.com; 11 Fundação Oswaldo Cruz, Instituto Leônidas e Maria Deane, Fiocruz Amazônia, Manaus 69057-070, Amazonas, Brazil; stefanie.lopes@fiocruz.br; 12 Laboratório de Princípios Ativos da Amazônia, Instituto Nacional de Pesquisas da Amazônia, Manaus 69067-375, Amazonas, Brazil; smnunomu@gmail.com

**Keywords:** Amazon, malaria, *Plasmodium*, *Piper*, ishwarane, substance

## Abstract

**Background/Objectives**: In the Brazilian Amazon, which accounts for over 99% of national malaria cases, 34,260 cases were reported as of August 2025, predominantly caused by *Plasmodium vivax*, responsible for 86.69% of the infections. The increasing resistance of the parasite to conventional therapies highlights the urgent need for novel control strategies, with essential oils and plant-derived substances emerging as promising alternatives. **Methods**: In this context, we evaluated the anti-*Plasmodium* potential of *Piper alatipetiolatum* essential oil and its major constituent 6-ishwarone against *P. vivax*, including cytotoxicity in Vero and PBMCs, molecular docking on dihydrofolate reductase (DHFR) and lactate dehydrogenase (LDH), and in silico pharmacokinetic profiling. **Results**: Both the oil and 6-ishwarone inhibited *P. vivax* dose-dependently (2.1 ± 1 to 100%), with IC_50_ values of 9.25 µg/mL and 3.93 µg/mL, respectively. Importantly, no cytotoxic effects were observed at 24 h, with cell viability ranging from 94.7% to 98.3%, highlighting the selectivity of these compounds towards the parasite over mammalian cells. Docking studies indicated selective binding of 6-ishwarone to DHFR (−7.7 kcal/mol; Ki = 2.27 µM) with key interactions (Trp816, Lys820, Tyr819, Asn823, Thr865), whereas binding to LDH was weaker (−6.2 kcal/mol; Ki = 28.10 µM), suggesting DHFR as the primary molecular target. In silico ADMET predictions and experimental data indicated favorable drug-like properties: TPSA = 20.23 Å^2^, moderate lipophilicity (LogP = 3.37), soluble (ESOL Log S = −3.58; Ali Log S = −3.89; Silicos-IT Log S = −2.84), high gastrointestinal absorption, BBB permeability (0.985), not a P-glycoprotein substrate (0.11), and low likelihood of CYP inhibition. Toxicity predictions showed non-mutagenic and non-hepatotoxic effects, low cardiotoxicity (hERG inhibition risk 0.08–0.32), low reproductive toxicity (0.03), moderate neurotoxicity (0.28), low acute toxicity (oral LD_50_ = 2.061 mol/kg), and low chronic toxicity (LOAEL = 1.995 log mg/kg/day). **Conclusions**: Together, these findings demonstrate that essential oil and 6-ishwarone of *P. alatipetiolatum* are selective, bioavailable, and promising natural leads for antimalarial drug development.

## 1. Introduction

Malaria is an infectious disease that remains a significant public health challenge in tropical and subtropical regions, causing over 200 million cases and around 500,000 deaths each year [1]. In fact, in 2024, the global number of malaria cases reached an estimated 263 million, corresponding to an incidence of 60.4 cases per 1000 people at risk, which represents an increase of 11 million cases compared with 2023 when the incidence was 58.6 per 1000, and the World Health Organization (WHO) reports that the African Region continues to bear the heaviest burden, accounting for approximately 94% of all malaria cases worldwide [2].

Within the Americas, 505,642 malaria cases were reported in 2024, with five countries accounting for almost 90% of the cases, namely Brazil (33%), the Bolivarian Republic of Venezuela (26%), Colombia (21%), Guyana (6%), and Peru (4%) [3]. In particular, in Brazil, the Amazon Region is considered endemic for malaria, representing over 99% of the country’s autochthonous cases, with 34,260 cases reported by August 2025, of which 44.7% and 37.95% occurred in indigenous and rural areas, respectively, highlighting the persistent regional challenge [4].

Malaria is caused by parasites of the genus *Plasmodium* (Plasmodiidae), with five species capable of infecting humans, among which *P. falciparum* Welch, 1897 and *P. vivax* Grassi & Feletti, 1890 represent the greatest threat, as *P. falciparum* is the most deadly and prevalent parasite in Africa while *P. vivax* dominates in most countries outside the continent, and the other species that can infect humans include *P. malariae* Laveran, 1881, *P. ovale* Stephens, 1922 and *P. knowlesi* (Stephens & Christophers, 1931), with global cases of *P. knowlesi* increasing by 18.9% in 2024, totalling 3290 reported cases and indigenous cases rising by 22% compared with the previous year [3].

Specifically in Brazil, *P. falciparum* and *P. vivax* are responsible for 13.31% and 86.69% of malaria cases, respectively, underscoring the importance of targeted disease control efforts [4], and the infection is transmitted primarily by mosquitoes of the genus *Anopheles* (Culicidae), particularly *An. darlingi* Root, 1926, which serves as the main vector in the region [5].

Nevertheless, secondary vector species such as *An. nuneztovari* Gabaldón, 1940, *An. triannulatus* Neiva & Pinto, 1922, *An. albitarsis* Lynch Arribálzaga, 1878, *An. aquasalis* Curry, 1932, and *An. braziliensis* Chagas, 1907 can also contribute to malaria transmission [6,7], with their vectorial capacity influenced by ecological, genetic, and behavioural factors including host preference, longevity, and population density, particularly in specific ecological settings or in areas where *An. darlingi* is absent or occurs at low densities [7]. Control of these vectors remains a significant challenge due to widespread resistance to various synthetic insecticides, particularly pyrethroids, reported in numerous studies [3,8,9].

Beyond vector management, a further critical obstacle to effective malaria control is the emergence and spread of drug resistance in *Plasmodium* parasites, which severely compromises treatment efficacy, with resistant strains of *P. falciparum* and, increasingly, *P. vivax* reducing the therapeutic lifespan of frontline antimalarials [4]. Resistance is commonly monitored through shifts in the half-maximal inhibitory concentration (IC_50_) of drugs, where elevated values indicate declining parasite susceptibility.

For instance, *P. falciparum* and *P. vivax* isolates resistant to pyrimethamine often display IC_50_ values exceeding 0.025 µg/mL, compared with sensitive strains typically below 0.0025 µg/mL. Similarly, sulfadoxine-resistant parasites can reach IC_50_ values over 0.31 µg/mL, reflecting near-complete loss of drug efficacy [10].

Central to these resistance mechanisms are mutations in pivotal enzymes of parasite metabolism, particularly those of the folate and glycolytic pathways [11]. Dihydrofolate reductase (DHFR) catalyses the reduction of dihydrofolate to tetrahydrofolate, an essential step in nucleotide synthesis and DNA replication, whereas inhibition of DHFR by pyrimethamine prevents DNA synthesis and parasite proliferation; however, mutations in the *dhfr* gene (e.g., S108N, N51I, C59R, I164L) diminish drug binding while preserving enzymatic function, thereby conferring resistance [12].

Another critical enzyme is lactate dehydrogenase (LDH), a key component of the parasite’s glycolytic pathway, which is the primary source of ATP for *Plasmodium* during intraerythrocytic stages [13]. Inhibition of LDH disrupts energy production, leading to metabolic collapse and parasite death [14]. Because LDH from *Plasmodium* differs structurally from the human counterpart, it represents an attractive therapeutic target, with several natural and synthetic inhibitors showing selective activity against the parasite enzyme [15]. Mutations in the *ldh* gene, although less extensively documented than in *dhfr*, may also reduce inhibitor binding and contribute to treatment failure [16].

When these enzymes are successfully inhibited, parasites experience nucleotide depletion, impaired energy metabolism, replication arrest, and eventual death, validating them as valuable therapeutic targets [17]. However, the accumulation of resistance-associated mutations in *dhfr* and, to a lesser extent, *ldh* illustrates the parasite’s remarkable adaptability and underscores the urgent need for continuous surveillance, rational drug design, and innovative therapeutic strategies [12].

In light of escalating resistance to conventional antimalarials, plant-derived substances ranging from essential oils to purified phytochemicals are attracting growing interest as alternative interventions against *Plasmodium* spp. [18]. Historically, some of the most effective antimalarial drugs, such as quinine from *Cinchona* spp. (Rubiaceae) bark and artemisinin from *Artemisia* spp. (Asteraceae), originated from medicinal plants, underscoring the value of phytochemicals as sources of novel therapeutic agents [11].

Essential oils, rich in terpenes and phenylpropanoids, extracted from several plants, represent one of the most explored classes of natural mixtures with antimalarial activity and their complex composition, typically dominated by terpenes and phenylpropanoids, enables multimodal mechanisms of action, which can reduce the likelihood of resistance development [14].

For instance, essential oil (0.2 to 2 µg/mL) from *Artemisia pallens* Wall. ex DC. (Asteraceae) (IC_50_ of 1.98 µg/mL) [19], *Cladanthus mixtus* (L.) Chevall. (Asteraceae), *Salvia sclarea* L. (Lamiaceae), Salvia *officinalis* L. (Lamiaceae), *Thymus vulgaris* L. (Lamiaceae), *Pinus mugo* Turra (Pinaceae), and *Chamaemelum nobile* (L.) All. (Asteraceae) (20 to 100 µg/mL) (IC_50_ from 16.12 to 44.16 µg/mL) [20] showed activity against *P. falciparum*, *P. vivax*, and *P. berghei* Vincke & Lips, 1948.

Beyond complex mixtures, isolated bioactive substances often deliver more potent activity. The alkaloid cryptolepine, derived from *Cryptolepis sanguinolenta* (Lindl.) Schltr, (Apocynaceae) exhibits IC_50_ values of 0.140 µg/mL against asexual blood stages of *P. falciparum* and 0.0115 µg/mL against mature gametocytes, though its major extract is even more potent but toxicity limits its clinical utility [21].

In addition to their antiparasitic effects, assessing the cytotoxicity of plant-derived products such as essential oils and isolated compounds is essential for evaluating therapeutic potential [21]. Tests on mammalian cell lines, including Vero cells and peripheral blood mononuclear cells (PBMCs), provide critical safety data and enable calculation of the selectivity index (SI), which reflects the balance between efficacy and toxicity [22,23]. A high SI indicates selective action against the parasite with minimal host cell damage, underscoring why cytotoxicity screening is indispensable in early natural product-based drug discovery [22].

Guided by these principles of safety and efficacy, our research focused on exploring Amazonian plant resources with anti-mosquito potential. In our previous studies, we investigated for the first time the essential oil of *Piper alatipetiolatum* Yunck. (Piperaceae), a species native to the Amazon rainforest and traditionally underexplored in terms of its phytochemical and biological potential.

The essential oil was obtained in 7.6 ± 0.1% yield, with a pH of 4.5 ± 0.8, refractive index of 1.57 ± 0.4, density of 0.921 ± 0.1 g/cm^3^, and colourless appearance. It was mainly composed of oxygenated sesquiterpenes (86.8%) and sesquiterpenes (12.2%), dominated by ishwarone (78.6%), followed by ishwarol B, β-elemene, selin-11-en-4α-ol, and ishwarane. Bioassays showed efficacy against *Aedes aegypti* Linnaeus, 1762 (Culicidae), with larvicidal, pupicidal, and ovicidal effects linked to disruption of detoxification pathways and acetylcholinesterase inhibition [23].

We reported for the first time the isolation of 6-ishwarone, an unprecedented ishwarane-type sesquiterpene and the major substance of essential oil from *P. alatipetiolatum*, obtained as white crystalline needles, with molecular formula C_15_H_22_O confirmed by EI-MS ([M]^+^ *m*/*z* 218) and HR-APCI-MS ([M + H]^+^ *m*/*z* 219.1745, calc. 219.1743). NMR data revealed a unique ishwarane skeleton with a carbonyl at C-6, distinguishing it from 3- and 8-ishwarone [24]. The substance showed potent larvicidal activity against *Ae. aegypti* and *An. darlingi*, the main malaria vector in the Amazon, with LC_50_ of 25 to 26 µg/mL. Mechanistic studies indicated induction of oxidative stress via overproduction of reactive oxygen and nitrogen species, modulation of detoxifying enzymes, thiol depletion, and strong acetylcholinesterase inhibition, leading to neuromuscular dysfunction and larval mortality [7].

The interest in 6-ishwarone lies primarily in its unprecedented chemical structure. This novel ishwarane-type sesquiterpene bears a carbonyl group at C-6, which distinguishes it from the previously known 3- and 8-ishwarone isomers commonly reported in *Piper* species [24]. This unique structural feature suggests distinct physicochemical properties and potential reactivity, making 6-ishwarone an attractive candidate for further studies on its biological and pharmacological activities [7].

Taken together, the chemical characterisation of *P. alatipetiolatum* essential oil and the unprecedented isolation of 6-ishwarone provide a solid foundation for future studies. Given its ability to disrupt redox balance and inhibit key enzymes, 6-ishwarone emerges as a promising candidate for evaluation against *P. vivax*. Its predicted mechanism of action can be further explored through molecular docking on relevant enzymatic targets, alongside in silico pharmacokinetic and ADMET analyses to assess binding interactions, bioavailability, and drug-like properties prior to in vivo testing. These findings highlight its potential as a novel natural scaffold for anti-*Plasmodium* drug development.

## 2. Material and Methods

### 2.1. Reagents and Cell Lines

All reagents used in this study were of analytical grade and purchased from Sigma-Aldrich (St. Louis, MO, USA), including dimethyl sulfoxide (DMSO), MTT reagent (3-(4,5-dimethylthiazol-2-yl)-2,5-diphenyl tetrazolium bromide), Ficoll-Hypaque, and the culture media IMDM (Iscove’s Modified Dulbecco Medium), RPMI (Roswell Park Memorial Institute medium), DMSO, and DMEM (Dulbecco’s Modified Eagle Medium). Supplements included human AB serum, fetal bovine serum (FBS), penicillin–streptomycin, and fungizone. The Vero CCL-81 cell line was derived from the kidney of the African green monkey *Chlorocebus sabaeus*.

### 2.2. Plant Procedures

All procedures for collecting *P. alatipetiolatum* leaves, including taxonomic identification, voucher specimen deposition, air-drying at room temperature, and the respective legal authorizations issued by the National System for the Management of Genetic Heritage and Associated Traditional Knowledge and the Authorization and Information System on Biodiversity, are described in detail in our study [23].

### 2.3. Essential Oil Extraction and Chromatographic Isolation of 6-Ishwarone

The essential oil extraction procedures subsequently analysed by gas chromatography mass spectrometry and gas chromatography with flame ionization detection, as well as the isolation of 6-ishwarone are thoroughly detailed in our recent publications [7,9].

### 2.4. Collection of Peripheral Blood from Volunteers Infected with P. vivax

Adult volunteers aged 18 years or older presenting to the Fundação de Medicina Tropical Dr. Heitor Vieira Dourado were screened for *P. vivax* infection. Diagnosis and species identification were performed by thick and thin blood smears stained with Giemsa and examined by experienced microscopists according to the Brazilian Ministry of Health guidelines [25]. Only samples confirmed as *P. vivax* positive, with parasitemia equal to or greater than two crosses, corresponding to 501 to 10,000 parasites per µL, were included in the study. All participants provided written informed consent prior to enrolment. Approximately 9 mL of peripheral blood were collected by venipuncture into sterile, heparinized Vacutainer^®^ (Becton, Dickinson and Company, Franklin Lakes, NJ, USA) tubes from each participant.

### 2.5. Assessment of Inhibitory Activity Against P. vivax

The ex vivo maturation assay was conducted following the established methodology for *P. vivax*, with modifications [25]. The blood pellet containing infected erythrocytes was resuspended in Iscove’s Modified Dulbecco Medium (IMDM) supplemented with 20% human AB serum to achieve a 2% hematocrit. Aliquots of 200 µL of this suspension were dispensed into a 96-well plate.

The essential oil and 6-ishwarone were solubilised in 1 mL of DMSO to prepare stock solutions at 10 mg/mL and were added to the wells to achieve final concentrations of 1, 3, 6, 12, 25, 50, and 100 µg/mL for both treatments. Chloroquine was used as a positive control at the same concentrations. An untreated group served as the negative control. All assays were performed in triplicate.

Plates were incubated for up to 48 h at 37 °C in a controlled gaseous atmosphere of 5% CO_2_, 1% O_2_, and 94% N_2_. Parasitemia was assessed by preparing thick blood smears stained with 5% Giemsa solution for 30 min and examined microscopically. The number of normal schizonts was determined by counting 200 parasites per slide.

### 2.6. Evaluation of Cell Viability in PBMC and VERO

The cytotoxicity of the essential oil and 6-ishwarone was assessed using the MTT assay (3-(4,5-dimethylthiazol-2-yl)-2,5-diphenyl tetrazolium bromide) according to Mosmann [26], with adaptations following Niksic et al. [27]. Peripheral blood mononuclear cells (PBMCs) were isolated by density gradient centrifugation using Ficoll-Hypaque (GE Healthcare, Chicago, IL, USA) and washed three times in RPMI medium before being cultured.

PBMCs (5 × 10^5^ cells/well) and Vero cell lines (1 × 10^5^ cells/well) were seeded into 96-well plates containing 200 µL of RPMI or DMEM medium, respectively, both supplemented with 10% foetal bovine serum (FBS), penicillin–streptomycin, and fungizone. Cells were incubated at 37 °C in a humidified atmosphere containing 5% CO_2_ for 24 h to allow adherence and formation of sub-confluent monolayers.

After this period, the cells were treated with the essential oil and 6-ishwarone at concentrations of 3, 6, 12, 25, 50, and 100 µg/mL, and incubated again under the same conditions for 24, 48, and 72 h. Untreated cells were used as the negative control, and 1 mL of DMSO was included as the positive control.

At each time point, the culture medium was removed, and 10 µL of MTT solution (5 mg/mL in sterile PBS), diluted in 100 µL of phenol red–free DMEM, was added to each well to avoid absorbance interference. Plates were incubated for 4 h at 37 °C in 5% CO_2_. After incubation, the MTT solution was discarded and replaced with 50 µL of MTT lysis buffer. The plates were gently agitated to dissolve the formazan crystals and incubated for a further 10 min at 37 °C.

The optical density was measured at a wavelength of 492 nm using a microplate reader. Cell viability was calculated as the percentage of absorbance in treated cells relative to untreated controls using the equation: (A_492_ treated/A_492_ untreated) × 100.

### 2.7. Docking Molecular

The molecular docking simulations were carried out in accordance with the protocol described by Lima et al. [28]. The three-dimensional structure of 6-ishwarone was constructed, and its protonation state at physiological pH (7.4) as well as potential tautomeric forms were evaluated using Marvin Sketch. The resulting structure was then energy-minimised employing the PM7 semi-empirical method implemented in MOPAC2016, in order to obtain its lowest-energy conformation. The optimised molecule was subsequently converted to PDBQT format using AutoDock Tools version 1.5.6 [29].

The crystal structure of *P. vivax* dihydrofolate reductase (PvDHFR; PDB ID: 2BL9, 1.90 Å) co-crystallised with the inhibitor 5-(4-chlorophenyl)-6-ethylpyrimidine-2,4-diamine (CP6) was retrieved from the RCSB Protein Data Bank. Similarly, the crystal structure of lactate dehydrogenase (LDH; PDB ID: 1T2D, 1.10 Å) co-crystallised with Nicotinamide Adenine Dinucleotide (NAD) was also obtained.

For both targets, receptor preparation followed standard procedures, and the docking grid was centred on the native co-crystallised ligand to fully encompass the enzyme’s active site. The grid box for PvDHFR was centred at x = 86.816, y = 3.392, z = 39.919 for the reference inhibitor, and at x = 54.893, y = 4.820, z = 39.919 for 6-ishwarone. For lactate dehydrogenase, the grid was centred at x = 32.8235, y = 17.1395, z = 10.2605 for NAD and at x = 25.511, y = 16.454, z = 3.630 for 6-ishwarone. For all docking simulations, the parameters were set as follows: exhaustiveness = 30, number of modes = 30, and energy range = 30.

All docking calculations were performed using AutoDock Vina, and the resulting binding poses were analysed and visualised using Discovery Studio Visualiser version 2021. To validate the docking protocol, the co-crystallised inhibitors were re-docked under identical conditions, and the root mean square deviation (RMSD) was determined. Furthermore, the inhibition mechanism and the steady-state inhibition constant (Ki) were calculated using Lineweaver–Burk plots, following the methodology outlined by Lima et al. [28].

### 2.8. In Silico Pharmacokinetic, Metabolism, and Toxicity Studies

Different pharmacokinetic and toxicity-related parameters, including Absorption, Distribution, Metabolism, Excretion, and general Toxicity (ADMET) were predicted in silico to support the interpretation of the molecular docking results and to provide a preliminary assessment of the drug-likeness of 6-ishwarone.

The analysis encompassed key descriptors including molecular weight, lipophilicity (LogP), solubility (LogS), bioavailability score, gastrointestinal absorption, intestinal permeability (Caco-2), volume of distribution (VDss), blood–brain barrier (BBB) penetration, interaction with P-glycoprotein (P-gp), and potential metabolism or inhibition of CYP450 enzymes. All predictions were performed using SwissADME and pkCSM [30,31]. These data provide complementary insights to the molecular docking simulations and contribute to a better understanding of the pharmacological potential of the 6-ishwarone.

### 2.9. Statistical Analysis

All experiments were conducted in triplicate, and results are presented as mean ± standard deviation (SD). For the MTT assay on PBMCs and VERO cells, cell viability was calculated as the ratio of absorbance between treated and control cells. IC_50_ values along with their confidence intervals were determined by fitting dose–response curves using nonlinear regression in Prism 9.0 (GraphPad Software, San Diego, CA, USA).

For the *P. vivax* ex vivo maturation assay, normal schizonts were counted in 200 parasites per slide, and parasite growth inhibition percentages were calculated relative to the untreated controls. The resulting IC_50_ values were likewise estimated by nonlinear regression. Data normality was assessed with the Shapiro–Wilk test. In addition, the homogeneity of variances was verified through the Brown–Forsythe and Bartlett’s tests.

Differences between the IC_50_ values for the anti-*Plasmodium* activity were specifically evaluated by one-way ANOVA, followed by Tukey’s post hoc test, with statistical significance set at *p* < 0.05. For the molecular docking, binding energies and estimated Ki values were descriptively compared with those of co-crystallised reference ligands, without additional inferential testing [5,7].

## 3. Results

### 3.1. Collection of Peripheral Blood from Volunteers Infected with P. vivax

To assess the ex vivo anti-*Plasmodium* activity of the essential oil and 6-ishwarone, a total of ten fresh *P. vivax* isolates were collected from adult volunteers. Baseline parasitological parameters for these isolates are presented in Table 1. At the start of the assays, the median percentage of parasites at the ring stage was 68% (range: 52–87%), indicating that the majority of parasites were at an early developmental stage suitable for the maturation assay, which relies on monitoring the progression from ring forms to mature schizonts under controlled culture conditions.

The geometric mean parasitaemia was 9163 asexual parasites per µL demonstrating an adequate parasite density to ensure reliable counting and consistent quantification of growth inhibition across the different concentrations tested. After incubation, the mean schizont count at harvest was 46.87 (95% CI: 42.33–50.33), reflecting the expected level of parasite development in untreated control wells and confirming that the culture conditions supported normal maturation. The median assay duration was 46.1 h (range: 45–48 h), which is consistent with the typical timeframe required for *P. vivax* maturation in short-term culture systems.

These baseline characteristics provide confidence in the robustness and reproducibility of the ex vivo assay, ensuring that observed inhibitory effects can be attributed to the tested compounds rather than to variations in parasite stage, density, or viability.

### 3.2. Assessment of Inhibitory Activity Against P. vivax

**Figure 1:**
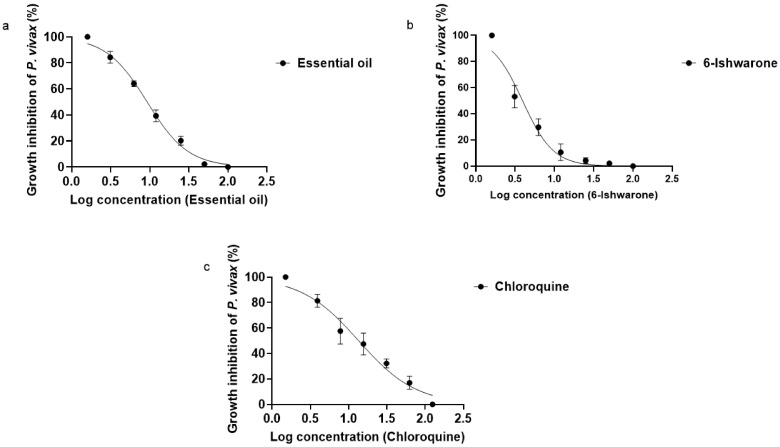
Dose–response curves for the inhibition of *P*. *vivax* by essential oil from *P*. *alatipetiolatum* (**a**), 6-ishwarone (**b**), and chloroquine (**c**). 2 Parasite cultures were treated with increasing concentrations of each, and parasite growth inhibition (%) was measured. Data were fitted using a 3 nonlinear regression to determine IC_50_ values. Results represent the mean of three independent experiments performed in triplicate.

The essential oil from *P. alatipetiolatum*, 6-ishwarone, and chloroquine exhibited dose-dependent inhibition profiles against *P. vivax*, with statistically robust fits to sigmoidal curves generated by four-parameter non-linear regression. However, marked differences were observed in terms of potency and response dynamics among the treatments (Figure 1a–c).

6-Ishwarone proved to be the most potent treatment, with an IC_50_ of 3.93 µg/mL and a steep dose–response slope (HillSlope = −2.217), indicating a highly sensitive response to concentration changes. Parasite inhibition ranged from 47.9 ± 3 to 100%, with pronounced increases occurring even at small dose increments. This behaviour suggests that minor variations in concentration result in sharp changes in inhibitory effect, a desirable feature for compounds with therapeutic applications. The dose–response curve showed excellent fit to the experimental data (R^2^ = 0.9679).

The essential oil showed an IC_50_ of 9.25 µg/mL, with a less steep slope (HillSlope = −1.651), potentially reflecting the combined effect of multiple constituents with varying levels of biological activity. Inhibition ranged from 2.1 ± 1 to 100%, following a more gradual pattern compared to the isolated compound. Notably, 6-ishwarone accounts for 78% of the essential oil chemical composition, according to chromatographic analyses [32], supporting the hypothesis that its high concentration is a key determinant of the observed anti-*Plasmodium* activity. The model also showed a robust fit (R^2^ = 0.9932), further corroborating its biological efficacy.

Chloroquine, used as a positive control, exhibited an IC_50_ of 13.53 µg/mL and the lowest dose–response slope among the treatments (HillSlope = −1.155), indicating a more gradual inhibition profile in response to increasing concentrations. Inhibition ranged from 16.9 ± 1 to 100%, demonstrating effectiveness even at lower doses, although with lower inhibitory potency compared to the essential oil and 6-ishwarone, the model exhibited a good fit (R^2^ = 0.9739), consistent with its known pharmacological activity.

Statistical analysis of the IC_50_ values confirmed significant differences among treatments (ANOVA: F (2, 6) = 10.25; *p* = 0.0116; R^2^ = 0.7736), and tests for variance homogeneity (Brown–Forsythe and Bartlett) indicated no significant differences between variances (*p* > 0.05), validating the use of the model. Tukey’s multiple comparisons test revealed that the IC_50_ of 6-ishwarone was significantly lower than that of chloroquine (*p* = 0.0095), confirming its superior potency. However, no statistically significant differences were found between 6-ishwarone and the essential oil (*p* = 0.1144), nor between the essential oil and chloroquine (*p* = 0.1671) (Table 2).

These findings demonstrate that both the essential oil and 6-ishwarone possess significant anti-*Plasmodium* activity, with the isolated substance outperforming chloroquine in terms of potency. The high concentration of 6-ishwarone within the essential oil supports the hypothesis that this substance is chiefly responsible for the observed biological effect, positioning it as a promising candidate for the development of new antimalarial therapies.

### 3.3. Evaluation of Cell Viability in PBMC and VERO

**Figure 2:**
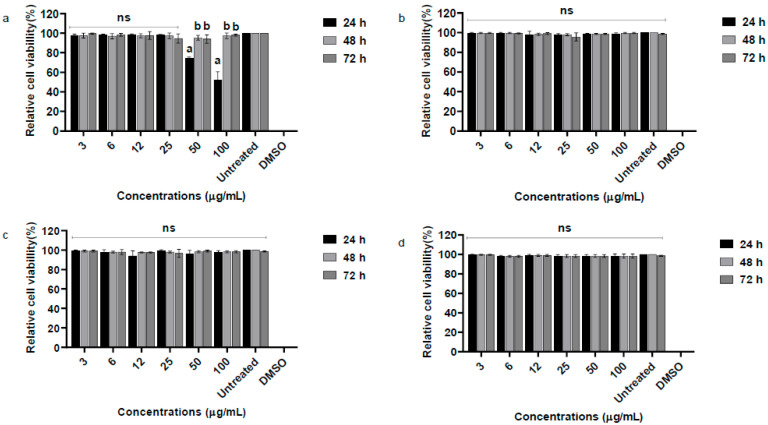
Relative cell viability (%) of Vero and PBMC cells after exposure to the essential oil of *P*. *alatipetiolatum* and 6-ishwarone at concentrations ranging from 3 to 100 μg/mL for 24, 48, and 72 h. Panels (**a**,**b**) show the results for Vero and PBMC cells, respectively, treated with the essential oil, while panels (**c**,**d**) correspond to Vero and PBMC cells treated with 6-ishwarone. The MTT assay was used to assess cytotoxicity, and cell viability is expressed as a percentage relative to untreated controls. DMSO (100%) was included as a positive control. Data are shown as mean ± standard deviation (*n* = 3). Statistical analysis was performed using one-way ANOVA followed by Tukey’s post hoc test. Different letters 10 indicate statistically significant differences between groups (*p* < 0.05); ns = not significant.

Cell viability of VERO cells and PBMCs treated with essential oil from *P. alatipetiolatum* was evaluated using the MTT assay at three time points (24, 48, and 72 h) and concentrations ranging from 3 to 100 µg/mL (Figure 2a,b). In VERO cells, the essential oil exhibited low cytotoxicity up to 25 µg/mL, with viability ranging from 94.7 ± 9% to 98.3 ± 6% at 24 h.

At higher concentrations, significant reductions were observed at 24 h, with mean viabilities of 74.0 ± 1.7% and 52.3 ± 8.3% at 50 and 100 µg/mL, respectively. Notably, viability recovered at later time points, suggesting possible cellular adaptation. Statistical analysis demonstrated highly significant effects of concentration (93.12% of variance; F (7, 48) = 1395.49; *p* < 0.0001), time (1.28% of variance; F (2, 48) = 67.12; *p* < 0.0001), and their interaction (5.14% of variance; F (14, 48) = 38.50; *p* < 0.0001) on VERO cell viability.

In PBMCs, the essential oil did not significantly affect viability, which remained close to 99.7 ± 1.2% at 100 µg/mL after 24 h. Only concentration had a significant influence (99.88% of variance; F (7, 48) = 6778.16; *p* < 0.0001), while time (0.27%; F (2, 48) = 0.65; *p* = 0.5268) and interaction (0.02%; F (14, 48) = 0.71; *p* = 0.7553) were not significant.

Similarly, VERO cells and PBMCs treated with 6-ishwarone showed high viability at all tested concentrations and time points (Figure 2c,d). VERO cell viability remained generally above 94.5 ± 3%, while PBMC viability was around 98.0 ± 1%. Two-way ANOVA confirmed that only time had a significant effect (VERO: F (7, 48) = 2889.81; *p* < 0.0001; PBMC: F (7, 48) = 7670.46; *p* < 0.0001), whereas concentration and interaction were not significant, indicating minor temporal variations independent of dose. Positive and negative controls confirmed assay reliability.

These findings support a favorable in vitro safety profile for 6-ishwarone and indicate that, while the essential oil warrants further mechanistic and in vivo toxicity evaluation at higher doses, both substances are suitable for continued pharmacological investigation.

### 3.4. Docking Molecular

The molecular docking analysis was conducted to evaluate the inhibitory potential of 6-ishwarone against two target enzymes of *P. vivax*: dihydrofolate reductase (DHFR) and lactate dehydrogenase (LDH), compared to the standard ligands CP6 and NAD, respectively (Table 3 and Table 4).

Regarding DHFR (PDB: 2BL9), 6-ishwarone exhibited a binding energy of −7.7 kcal/mol and an estimated inhibition constant (Ki) of 2.27 µM, demonstrating higher affinity than the reference compound CP6 (−7.2 kcal/mol; Ki = 5.30 µM). The primary interactions involved Pi–Alkyl and Alkyl bonds with residues Trp816, Lys820, and Tyr819, as well as van der Waals contacts and hydrogen bonds with Asn823 and Thr865, which stabilise the ligand’s carbonyl group. Interaction distances ranged between 3.16 and 4.55 Å for hydrophobic bonds, and approximately 2.7 Å for hydrogen bonds, suggesting a stable orientation within the active site.

**Figure 3:**
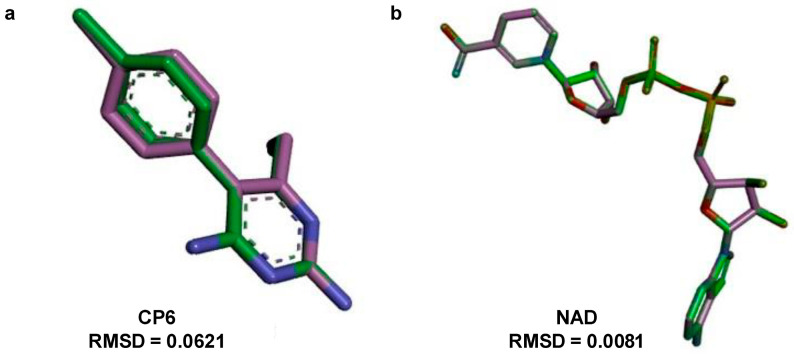
Superposition and root-mean-square deviation (RMSD) analysis of docked conformations. (**a**) Structural alignment of CP6 [5-(4-chlorophenyl)-6-ethylpyrimidine-2,4-diamine] docked to dihydrofolate reductase from *P*. *vivax* (PDB: 2BL9), showing an RMSD of 0.0621 Å, indicating high accuracy of the docking pose relative to the reference structure. (**b**) Structural alignment of NAD (Nicotinamide-Adenine-Dinucleotide) and lactate dehydrogenase from *P*. *vivax* (PDB: 1T2D), with an RMSD of 0.0081 Å, confirming excellent conformational overlap. The green stick models represent the docked ligand, while the purple stick models indicate the reference conformer used for RMSD calculation.

**Figure 4:**
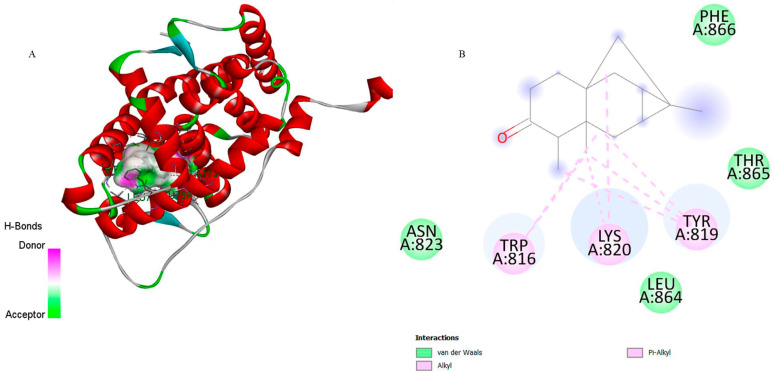
3D representation of the molecular docking between 6-ishwarone and dihydrofolate reductase from *P*. *vivax* (PvDHFR). (**A**) The binding pocket shows 6-ishwarone forming Pi–Alkyl, Alkyl interactions with Trp816, Lys820, and Tyr819, van der Waals contacts with Asn823, Leu864, Thr865, and Phe866, and additional hydrogen bonds with Asn823 and Thr865 stabilizing the carbonyl moiety of the ligand. (**B**) The 2D interaction map confirms these hydrophobic and polar contacts and highlights the key distances involved in the ligand–enzyme binding mode.

Figure 3 corroborates these findings, wherein the structural superimposition with the standard ligand (Figure 3a) shows an RMSD of 0.0621 Å, indicating high accuracy of the docking pose; meanwhile, Figure 4 presents the 3D and 2D spatial distribution of 6-ishwarone’s interactions with key residues, confirming the presence of essential hydrophobic and polar contacts for complex stability.

**Figure 5:**
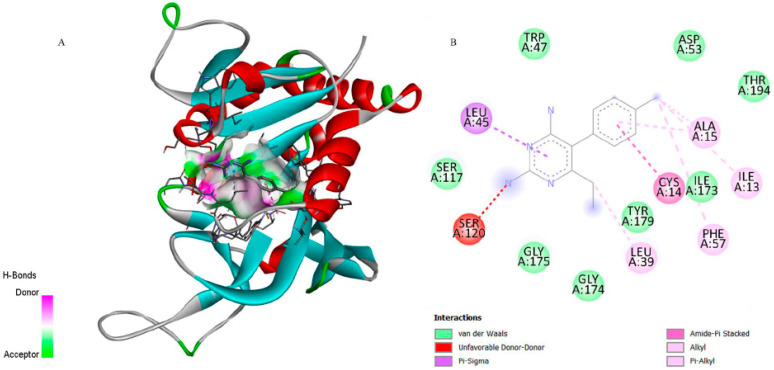
3D representation of the molecular docking between CP6 [5-(4-chlorophenyl)-6-ethylpyrimidine-2,4-diamine] and dihydrofolate reductase from *P. vivax* (PvDHFR). (**A**) Close-up view of the binding pocket showing CP6 interacting with residue Ser120 through an unfavorable donor–donor interaction, Leu45 through a Pi–Sigma interaction, and Leu39, Ala15, Ile13, Ile173, Tyr179, Phe57, and Cys14 through alkyl or Pi–alkyl contacts stabilizing the ligand within the active site. (**B**) 2D interaction map confirms the presence of these hydrophobic contacts and highlights additional van der Waals interactions with Trp47, Asp53, Thr194, Ser117, Gly174, and Gly175, supporting the ligand–enzyme binding mode.

Although CP6 interacted with a greater number of residues (Figure 5), it established an unfavourable donor–donor interaction with residue Ser120, which may have contributed to its lower affinity. This qualitative difference in interaction types is supported by the lower Ki of 6-ishwarone, suggesting that despite fewer total interactions, those formed are energetically more favourable.

**Figure 6:**
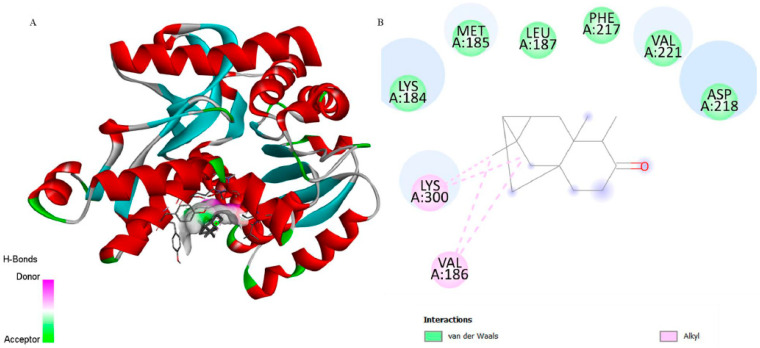
3D representation of the molecular docking between 6-ishwarone and lactate dehydrogenase from *P*. *vivax*. (**A**) The binding pocket shows 6-ishwarone forming Alkyl interactions with Val186 and Lys300; van der Waals contacts with Lys184, Met185, Leu187, Phe217, Val221, and Asp218; and additional hydrogen bonds with surrounding residues, stabilizing the ligand within the active site. (**B**) The 2D interaction map confirms these hydrophobic and polar contacts, including Alkyl interactions, and highlights the detailed view of the ligand–receptor binding mode.

Concerning LDH (PDB: 1T2D), 6-ishwarone exhibited a binding energy of −6.2 kcal/mol and a Ki of 28.10 µM, revealing considerably lower affinity compared to the endogenous ligand NAD (−11.3 kcal/mol; Ki = 5.19 µM). The main interactions of 6-ishwarone included Alkyl contacts with Val186 and Lys300, as well as van der Waals interactions with Lys184, Met185, Leu187, Phe217, and Asp218. Bond distances ranged between 2.2 and 2.7 Å, typical of moderate hydrophobic interactions (Figure 6).

**Figure 7:**
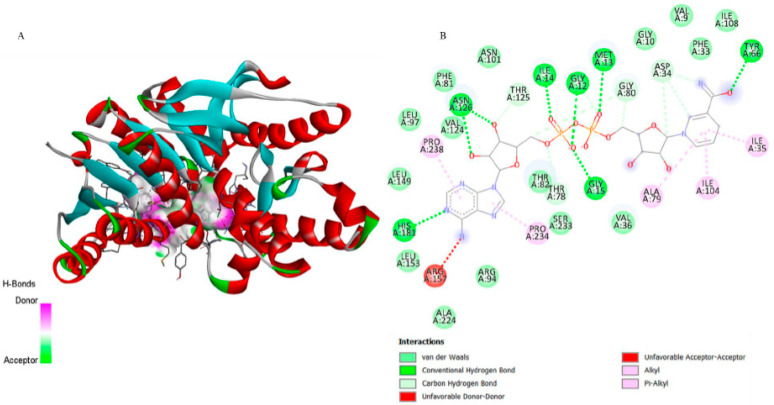
3D representation of the molecular docking between NAD (Nicotinamide-Adenine-Dinucleotide) and lactate dehydrogenase from *P*. *vivax*. (**A**) The binding pocket shows NAD forming Pi–Alkyl and Alkyl interactions with Phe81 and Arg157; van der Waals contacts with Asn101 and surrounding residues; and additional hydrogen bonds with Thr125 and Asn101, stabilizing the ligand within the active site. (**B**) The 2D interaction map confirms these hydrophobic and polar contacts, including conventional and carbon hydrogen bonds, and highlights unfavorable donor–donor and acceptor–acceptor proximities, providing a detailed view of the ligand–enzyme binding mode.

The superimposition with NAD (Figure 3b) showed an RMSD of only 0.0081 Å, validating the simulation’s precision. Figure 5 illustrates that although 6-ishwarone occupies the catalytic site, its interactions are less specific and primarily based on hydrophobic contacts. In contrast, NAD (Figure 7) formed an extensive network of hydrogen bonds with key residues (Thr125, Asn101, Gly12, among others), in addition to Pi–Alkyl interactions and H–C=O type contacts, resulting in a highly stable complex.

Taken together, the results indicate that 6-ishwarone possesses significant affinity for *P. vivax* DHFR, surpassing the standard CP6 in binding energy and lacking unfavourable interactions. Conversely, its low affinity for LDH compared to NAD suggests that this enzyme is unlikely to be its primary molecular target. These findings suggest that 6-ishwarone may interact with DHFR, supporting its potential involvement in the experimentally observed anti-*Plasmodium* activity.

### 3.5. In Silico Pharmacokinetic, Metabolism, and Toxicity Studies

An integrated analysis of the ADMET properties of 6-ishwarone was conducted, based on predictions from the SwissADME, pkCSM, and admetSAR platforms, complemented by in vitro experimental data. 6-ishwarone, with the molecular formula C_15_H_24_O and a molecular weight of 220.35 Da, has a topological polar surface area (TPSA) of 20.23 Å^2^ and no rotatable bonds, features that contribute to its structural rigidity. The consensus LogP is 3.37, indicating moderate lipophilicity.

Solubility predictions were: ESOL Log S = −3.58, Ali Log S = −3.89, and Silicos-IT Log S = −2.84, classifying the compound as soluble. These parameters suggest a good balance between permeability and solubility, which is favourable for oral absorption.

Gastrointestinal absorption was predicted to be high, and blood–brain barrier permeability was confirmed, indicating potential to reach the central nervous system. 6-Ishwarone is not a substrate of P-glycoprotein (P-gp), reducing the likelihood of efflux and associated poor absorption.

Regarding cytochrome P450 enzymes, the compound is not predicted to inhibit CYP1A2, CYP2D6, or CYP3A4, but it is expected to inhibit the CYP2C19 and CYP2C9 isoenzymes, which may suggest a potential for drug–drug interactions involving these enzymes. No PAINS (Pan-Assay Interference Compounds) or Brenk alerts were identified, although two lead-likeness violations were observed. The predicted bioavailability score was 0.55, consistent with moderate oral absorption.

Toxicological assessments indicated that 6-ishwarone is non-mutagenic, as evidenced by a negative Ames test result, and is not hepatotoxic. No inhibition of cardiac hERG I or II channels was predicted, suggesting a low risk of cardiotoxicity. The estimated oral lethal dose (LD_50_) in rats was 2.061 mol/kg, indicating low acute toxicity.

The lowest observed adverse effect level (LOAEL) for chronic toxicity was predicted to be 1.995 log mg/kg body weight per day. Skin sensitisation was predicted. Environmentally, the compound showed low toxicity towards *Tetrahymena pyriformis* (Tetrahymenidae) (0.575 log µg/L) and fish (0.681 log mM).

The admetSAR platform confirmed high permeability in Caco-2 cells (0.943) and very high human intestinal absorption (0.946), supporting a favourable absorption profile. The probability of BBB penetration was estimated at 0.985. The likelihood of being a P-gp inhibitor or substrate was low (0.40 and 0.11, respectively).

The risk of rodent carcinogenicity was low (0.14), as was reproductive toxicity (0.03). Neurotoxicity was classified as moderate (0.28), and the risk of skin sensitisation was considered high (0.66), in agreement with pkCSM data. The risk of cardiotoxicity, assessed via hERG channel inhibition, remained low, ranging from 0.08 to 0.32.

## 4. Discussion

The extraction of the essential oil from *P. alatipetiolatum* yielded 7.52 ± 1.4%, a relatively high value of both pharmacological and industrial significance. This elevated yield not only enhances the economic feasibility of extraction but also promotes sustainability in the utilisation of plant-derived raw materials and supports the potential for scale-up in production processes [33].

When compared to other *Piper* species commonly employed in phytochemical research, such as *P. purusanum* C.DC (4.2 ± 0.7%) [6], *P. brachypetiolatum* Yunck (1.5 ± 0.7%) [34], *P. tuberculatum* Jacq. (0.4 ± 0.1%) [35], and *P. baccans* (Miq) C.DC (2.2 ± 0.5%) [36], the yield obtained from *P. alatipetiolatum* is notably superior, reinforcing its potential as a viable source of bioactive compounds. The high extraction efficiency may also facilitate future pharmacological and preclinical studies by ensuring consistent availability of the active constituents [37].

The compound 6-ishwarone was isolated as needle-like crystals, with a molecular formula of C_15_H_22_O. Its chemical configuration, characterised by structural rigidity and lipophilicity, plays a pivotal role in its biological activity [7]. The conformational rigidity enhances specificity and stability against metabolic degradation during interactions with biological targets, while its lipophilic nature facilitates penetration through cellular membranes, promoting access to lipid-rich tissues [7].

Consequently, the combination of a high essential oil yield and the favourable structural features of 6-ishwarone contributes to a promising pharmacological profile, emphasising the importance of structural chemistry in the discovery of bioactive natural products and the development of novel plant-derived anti-*Plasmodium* agents [24,38].

In ex vivo assays against *P. vivax*, both the essential oil from *P. alatipetiolatum* and its major substance, 6-ishwarone, demonstrated clear dose-dependent inhibition, effectively suppressing parasite development from the ring to schizont stages [39]. This indicates that this substance can interfere with multiple critical stages of the parasite’s life cycle, a feature highly desirable for antimalarial candidates [40].

Notably, 6-ishwarone exhibited a remarkably low IC_50_ of 3.93 µg/mL and a steep Hill slope of −2.217, reflecting high sensitivity to concentration changes, where small variations in dose result in pronounced shifts in inhibitory effect. This steep dose–response relationship suggests potent and predictable anti-*Plasmodium* activity, advantageous for precise dosing in therapeutic applications [41]. By contrast, the whole essential oil, containing 78% 6-ishwarone, displayed a higher IC_50_ of 9.25 µg/mL with a shallower Hill slope of −1.651, indicative of a more gradual concentration–response curve [42]. This pattern implies that minor constituents within the essential oil may modulate the overall effect, either through additive or synergistic interactions, while the primary bioactivity remains attributable to 6-ishwarone [43].

These results collectively underscore that 6-ishwarone is the main bioactive agent driving the anti-*Plasmodium* effect, positioning it as a strong lead compound for further pharmacological development [44]. Moreover, its ability to inhibit multiple parasite stages suggests potential for improved therapeutic outcomes and a reduced likelihood of resistance development, reinforcing its value in antimalarial drug discovery [45].

Comparative literature data further support these findings. Essential oils from other *Piper* species, such as *P. claussenianum* C.DC, *P. aduncum* L., and *P. amalago* L., demonstrated anti-*Plasmodium* activity against *Plasmodium* spp., with IC_50_ values ranging from 8 to 26.51 µg/mL, reflecting moderate to strong activity depending on species and chemical composition [46]. Conversely, 70% hydroethanolic extracts from leaves, stems, and fruits of *P. retrofractum* L., *P. longum* L., *P. nigrum* L., and *P. betle* var. Tympew exhibited much lower activity, with IC_50_ values ranging from 500 to 1624.41 µg/mL [47].

When contextualised within a broader spectrum of plant-derived compounds, the potency of 6-ishwarone becomes particularly evident. For example, ellagic acid isolated from methanolic and ethanol/water extracts of *Mitragyna inermis* (Willd) O. Kuntze (Rubiaceae) exhibited strong anti-*Plasmodium* activity, with IC_50_ values of 0.84 and 1.62 µg/mL against *P. falciparum* and *P. vivax*, respectively [14], comparable to 6-ishwarone and highlighting the potential of phenolic structures in targeting *Plasmodium*.

Other phytochemicals, including α-mangostin, β-mangostin, γ-mangostin, punicalagin, and plumbagin isolated from several extracts from *Zingiber cassumunar* Roxb. (Zingiberaceae), *Punica granatum* L. (Lythraceae), *Garcinia mangostana* L. (Clusiaceae), *Alpinia galanga* (L.) Willd (Zingiberaceae), *Rhinacanthus nasutus* (L.) Kuntze (Acanthaceae), *Plumbago indica* L., (Plumbaginaceae), *Piper betle* L. (Piperaceae), Centella asiatica (L.) Urb. (Apiaceae), and *Senna tora* (L.) Roxb. (Fabaceae) displayed IC_50_ values ranging from 1.17 to 5.42 µg/mL, indicating moderate to strong activity. In contrast, substances such as asiaticoside, madecassic acid, and emodin showed moderate activity, with IC_50_ from 20.94 to 41.09 µg/mL. On the other hand, juglone, isoshinanolone, droserone, 6-gingerol, zingerone, asiatic acid, madecassoside, brazilin, protosappanin B, chrysophanol, rhein, and physcion exhibited low activity with IC_50_ values exceeding 50 µg/mL [13].

This comparison highlights that 6-ishwarone not only matches or surpasses the efficacy of potent plant-derived substances such as ellagic acid but also clearly outperforms many other phytochemicals commonly reported in the literature [14]. Moreover, when comparing the essential oil and isolated 6-ishwarone, it is evident that while minor constituents of the oil may modulate activity, the major anti-*Plasmodium* effect is driven by 6-ishwarone itself [47].

The variation in activity across different plant substances underscores the importance of both chemical composition and extraction methodology in determining anti-*Plasmodium* potential [48]. Importantly, 6-ishwarone’s pronounced activity at low micromolar concentrations, combined with its multi-stage inhibitory profile, positions it as a promising candidate for further pharmacological optimization, reinforcing its significance relative to other plant-derived antimalarial agents [39].

The exceptional anti-*Plasmodium* activity of the essential oil of *P. alatipetiolatum* and 6-ishwarone, naturally raises the question of its selectivity and safety toward mammalian cells [27]. While potent inhibition of *Plasmodium* is a critical attribute, the therapeutic value of any antimalarial candidate also depends on minimal cytotoxic effects on host cells [49]. Demonstrating high efficacy without compromising cell viability is therefore essential to ensure a favorable therapeutic window and to distinguish true anti-*Plasmodium* action from nonspecific cellular toxicity [50]

Equally important, the assessment of cytotoxicity in human PBMCs and VERO cells revealed a highly favourable safety profile for both essential oil and 6-ishwarone. Ishwarone maintained high cell viability, exceeding 94% in VERO cells and approximately 98% in PBMCs across all tested concentrations. The essential oil caused a transient reduction in VERO cell viability at 50 and 100 µg/mL, followed by recovery, suggesting activation of cellular adaptive mechanisms without irreversible damage. This lack of significant cytotoxicity is a crucial finding, as it indicates that the potent anti-*Plasmodium* activity observed is selective rather than a consequence of nonspecific toxicity [42].

The implications of this selectivity are substantial. Substances that indiscriminately affect host cells often possess a low clinical safety margin, limiting their therapeutic applicability [46]. In contrast, the essential oil and 6-ishwarone, which demonstrate strong activity against the parasite while sparing mammalian cells, offer a wider therapeutic window, enabling administration at effective doses without inducing adverse effects [44].

The high selectivity index (SI) recommended by the World Health Organization [51] preferably above 10 is a critical benchmark for the development of safe antimalarial candidates. In this study, both 6-ishwarone and the essential oil fulfilled this criterion, which further supports their potential in combination therapies, where low cytotoxicity could mitigate adverse effects and allow synergistic efficacy with existing antimalarials [52,53].

Beyond demonstrating potent and selective anti-*Plasmodium* activity, understanding the molecular mechanisms underlying 6-ishwarone’s efficacy is essential to fully contextualize its therapeutic potential. While ex vivo assays and cytotoxicity analyses confirmed both high activity against *P. vivax* and minimal effects on mammalian cells, mechanistic insights at the molecular level provide a rationale for these observations and highlight possible strategies to overcome drug resistance [54].

Molecular docking analyses provided such mechanistic insights, linking the compound’s molecular interactions with the observed suppression of *P. vivax* growth. 6-Ishwarone demonstrated high affinity for *P. vivax* dihydrofolate reductase (PvDHFR), with a binding energy of −7.7 kcal/mol and an estimated inhibition constant (Ki) of 2.27 µM.

Key interactions involved Pi–Alkyl and Alkyl contacts, as well as hydrogen bonds stabilising the carbonyl group of 6-ishwarone within the enzyme’s active site. DHFR catalyses the reduction of dihydrofolate to tetrahydrofolate, an essential cofactor for biosynthesis of purine and pyrimidine nucleotides, which are indispensable for DNA replication, RNA transcription, and protein synthesis [11]. By inhibiting PvDHFR, 6-ishwarone disrupts nucleotide biosynthesis, preventing DNA synthesis and halting the transition from ring to schizont stages, consistent with the ex vivo observations of parasite suppression [54].

The therapeutic relevance of dihydrofolate reductase (DHFR), particularly PvDHFR, is heightened in the context of drug resistance [55]. Mutations such as Ser-58 → Arg and Ser-117 → Asn significantly impair the efficacy of conventional antifolate drugs by introducing steric hindrance that disrupts inhibitor binding, although DHFR remains a classical target in antimalarial therapy, its clinical utility is increasingly limited due to widespread resistance [14].

Structural comparisons between wild-type PvDHFR and the pyrimethamine-resistant SP21 strain (bearing both Ser-58 → Arg and Ser-117 → Asn mutations), in complex with NADPH and either pyrimethamine (Pyr) or its analogue lacking the para-chlorine group (Pyr20), reveal that the Asn-117 side chain in the mutant enzyme causes steric conflict and weakens binding affinity [11]. This effect is further exacerbated by the loss of interaction with Ser-120, a residue critical for stabilising the inhibitor [14].

Previous studies have shown that mutations in *Plasmodium* DHFR conferring resistance to pyrimethamine often lead to cross-resistance to other classical antifolate inhibitors, such as cycloguanil, due to shared binding modes within the catalytic site [52,53]. However, the extent of this resistance depends on the structural characteristics of each compound, and inhibitors with distinct scaffolds like 6-ishwarone may retain affinity for resistant DHFR variants [44].

In contrast, 6-ishwarone demonstrates favourable binding characteristics, including stabilising hydrogen bonds and hydrophobic interactions, which may allow it to circumvent the steric limitations imposed by resistance-associated mutations, suggesting that 6-ishwarone retains potent activity even against DHFR variants that are resistant to traditional antifolate drugs [55,56].

On the other hand, 6-ishwarone exhibited low affinity for *P. vivax* lactate dehydrogenase (LDH) (−6.2 kcal/mol; Ki = 28.10 µM), indicating that this enzyme is unlikely to be the primary target of the compound [44]. These findings underscore that the selectivity of 6-ishwarone for DHFR is a critical determinant of its anti-*Plasmodium* activity, ensuring that the observed effects on the parasite are not due to nonspecific toxicity [55].

Lactate dehydrogenase (LDH) enzyme from *Plasmodium* species represents another critical molecular target, structurally and functionally distinct from human isozymes [11]. LDH catalyses the conversion of pyruvate into lactate during the final stage of glycolysis, a process essential for parasite energy production [13]. Hematin, a byproduct of hemoglobin digestion by malarial parasites, exerts toxicity by competing with NADH at the active site of *Plasmodium* [15].

Parasite survival depends on polymerizing hematin into hemozoin, which prevents its toxic effects within the food vacuole [49]. Antimalarial drugs such as chloroquine bind within the NADH binding pocket of LDH, occupying a position analogous to the adenyl ring of the cofactor and acting as competitive inhibitors of this crucial glycolytic enzyme [11]. These mechanistic insights suggest that 6-ishwarone may interfere with LDH function to some extent, potentially disrupting parasite energy metabolism while sparing host enzymes due to structural differences, although LDH is unlikely to be its primary target [50].

Overall, the molecular docking data provide a mechanistic explanation for the dose-dependent suppression of *P. vivax* growth observed ex vivo and highlight the potential of 6-ishwarone as a selective and safe candidate for the development of novel antimalarial agents targeting DHFR, including strains potentially resistant to conventional antifolate drugs [17,56].

Crucially, these mechanistic and safety insights are complemented by in silico analyses of the ADMET properties of 6-ishwarone, offering a comprehensive perspective on its drug-likeness [57]. Its physicochemical characteristics including moderate lipophilicity (LogP = 3.37), low topological polar surface area (TPSA = 20.23 Å^2^), and structural rigidity suggest high biological permeability and stability, favouring predictable interactions with molecular targets [58]. High predicted gastrointestinal absorption and intestinal permeability, along with potential blood–brain barrier penetration, indicate effective systemic distribution, reaching tissues that are otherwise difficult to access [47].

Importantly, the absence of cytotoxicity in VERO and PBMC cells, even at the highest tested concentrations, complements these ADMET predictions, confirming a wide therapeutic window: the concentrations required for potent anti-*Plasmodium* activity remain well below those that could compromise mammalian cell viability [59].

Regarding metabolism, 6-ishwarone is not predicted to inhibit major cytochrome P450 enzymes such as CYP3A4, CYP1A2, or CYP2D6, reducing the likelihood of clinically relevant drug–drug interactions [47]. While potential inhibition of CYP2C19 and CYP2C9 may warrant monitoring, the overall metabolic profile suggests manageable pharmacokinetics [47,60]. Toxicological predictions including a negative Ames test, absence of hepatotoxicity, and lack of hERG channel inhibition further reinforce its safety, aligning with the requirement for low off-target effects in antimalarial candidates [56].

Taken together, these pharmacokinetic, safety, and mechanistic characteristics indicate that 6-ishwarone combines high efficacy, selectivity, and safety with favourable systemic bioavailability and potential for oral administration [15,16]. In the context of increasing chloroquine resistance in *P. vivax*, these attributes position 6-ishwarone as a promising candidate for the development of novel, selective antimalarial agents, providing a strong foundation for further preclinical evaluation [16,61,62].

## 5. Conclusions

These findings demonstrate that the essential oil of *P. alatipetiolatum* and its major compound, 6-ishwarone exhibit strong and selective anti-*Plasmodium* activity, positioning them as promising candidates for novel antimalarial therapies. The potent inhibition of *P. vivax*, combined with low cytotoxicity in mammalian cells, supports the therapeutic potential of 6-ishwarone as a safe and effective natural substance. Moreover, molecular docking analyses suggest DHFR as a possible molecular target involved in the observed activity. Overall, this study highlights *P. alatipetiolatum* as a valuable source of bioactive sesquiterpenes, particularly 6-ishwarone, and reinforces the importance of natural products in combating drug-resistant malaria.

## Figures and Tables

**Table 1:** Baseline characteristics of *P. vivax* isolates used in the ex vivo maturation assay.

Characteristic	*P. vivax* (*n* = 10)
Median initial percentage of parasites at ring stage	68% (range: 52–87%)
Geometric mean parasitaemia (asexual parasites/µL)	9.163 (95% CI: 5.080–12.040)
Mean schizont count at harvest	46.87 (95% CI: 42.33–50.33)
Median assay duration (h)	46.1 (range: 45–48)

**Table 2:** IC_50_ Values of the essential oil and 6-ishwarone from *P. alatipetiolatum* against *P. vivax.*

Sample	IC_50_ (µg/mL) (LCL-UCL)	R^2^	df
Essential oil	9.25 ^ab^ (7.993 to 10.720)	0.9932	5
6-Ishwarone	3.93 ^b^ (3.051 to 5.134)	0.9679	5
Chloroquine	13.53 ^a^ (9.878 to 18.480)	0.9739	5

Half-maximal inhibitory concentration (IC_50_) values (µg/mL) for the essential oil, 6-ishwarone, and chloroquine against *P. vivax*, with corresponding 95% confidence intervals (LCL–UCL). R^2^ indicates the coefficient of determination for the nonlinear regression model fit, and df represents degrees of freedom used in the regression analysis. Different letters (a and b) in the same column indicate statistically significant differences among treatments, as determined by one-way ANOVA followed by Tukey’s post hoc test (F (2, 6) = 10.25, *p* = 0.0116).

**Table 3:** Comparative analysis of the inhibitory potential of 6-Ishwarone against dihydrofolate reductase from *P. vivax* compared to CP6.

Inhibitor	Interacting Residues	Chain	Bond Length (Å)	Type of Interaction	Binding Energy (kcal/mol)	Inhibition Constant (Ki µM)
CP6 *	Ser120	A	2.14	Unfavorable Donor-Donor	−7.2	5.30
	Leu45	A	3.75	Pi-Sigma		
	Leu39	A	3.95	Alkyl		
	Cys14	A	4.77	Pi-Alkyl		
	Ala15	A	4.58, 3.85	Pi-Alkyl		
	Ile13	A	4.59	Pi-Alkyl		
	Phe57	A	4.91	Pi-Alkyl		
	Tyr179	A	4.81	Pi-Alkyl		
	Ile173	A		van der Waals		
	Trp47	A		van der Waals		
	Asp53	A		van der Waals		
	Thr194	A		van der Waals		
	Ser117	A		van der Waals		
	Gly174	A		van der Waals		
	Gly175	A		van der Waals		
6-Ishwarone	Trp816	A	3.16	Pi-Alkyl	−7.7	2.27
	Lys820	A	3.08, 4.55	Pi-Alkyl, Pi-Alkyl		
	Tyr819	A	3.24, 3.84, 3.62	Pi-Alkyl, Pi-Alkyl, Alkyl		
	Ans823	A		van der Waals, H-bond		
	Thr865	A		van der Waals, H-bond		
	Leu864	A		van der Waals		
	Phe866	A		van der Waals		

* CP6: 5-(4-Chloro-phenyl)-6-ethyl-pyrimidine-2,4-diamine. Chain: indicates the specific protein chain (A) where the interaction occurs, according to the PDB structure used for the docking study.

**Table 4:** Comparative analysis of the inhibitory potential of 6-Ishwarone against lactate dehydrogenase from *P. vivax* compared to NAD.

Inhibitor	Interacting Residues	Chain	Bond Length (Å)	Type of Interaction	Binding Energy (kcal/mol)	Inhibition Constant (Ki µM)
NAD *	Ile14	A	1.98	Conventional H-Bond	−11.3	5.19
	Thr125	A	3.25	Conventional H-Bond		
	Val124	A	2.97	Conventional H-Bond		
	Asn126	A	2.48	Conventional H-Bond		
	Gly12	A	2.12, 2.11, 2.38, 2.78	Conventional H-Bond		
	Met13	A	2.15	Conventional H-Bond		
	Gly10	A	3.38	Conventional H-Bond		
	Gly80	A	3.31	Conventional H-Bond		
	Asp34	A	3.59	Conventional H-Bond		
	Thr82	A	3.58	Conventional H-Bond		
	Gly15	A	2.26, 2.26	Conventional H-Bond		
	Thr78	A	2.26	Conventional H-Bond		
	Ala79	A	3.39	Conventional H-Bond		
	Ile104	A	4.82	Conventional H-Bond		
	Ile35	A	3.81	Conventional H-Bond		
	Val36	A	3.81	Conventional H-Bond		
	Ser233	A	5.23	Conventional H-Bond		
	Pro234	A	5.23	Conventional H-Bond		
	His181	A	2.41	Conventional H-Bond		
	Arg157	A	7.25	Unfavorable-Acceptor-Acceptor		
	Pro238	A	5.36	Pi-Alkyl		
	Phe33	A	3.57	Pi-Alkyl		
	Phe81	A		Van der Waals		
	Asn101	A		Van der Waals		
	Leu149	A		Van der Waals		
	Leu97	A		Van der Waals		
	Leu153	A		Van der Waals		
	Ala224	A		Van der Waals		
	Arg94	A		Van der Waals		
6-Ishwarone	Lys300	A	2.72, 2.75	Alkyl	−6.2	28.10
	Val186	A	2.22, 2.29	Alkyl		
	Lys184	A		Van der Waals		
	Met185	A		Van der Waals		
	Leu187	A		Van der Waals		
	Phe217	A		Van der Waals		
	Val221	A		Van der Waals		
	Asp218	A		Van der Waals		

* NAD: Nicotinamide Adenine Dinucleotide. Chain: indicates the specific protein chain (A) where the interaction occurs, according to the PDB structure used for the docking study.

## Data Availability

The data presented in this study are available on request from the corresponding author.
